# In-Situ Growth of Graphene Films to Improve Sensing Performances

**DOI:** 10.3390/ma15217814

**Published:** 2022-11-05

**Authors:** Xinghong Liu, Liang Wu, Xiang Yu, Haoran Peng, Shijue Xu, Zilong Zhou

**Affiliations:** School of Materials Science and Technology, China University of Geosciences (Beijing), 29 Xueyuan Road, Haidian, Beijing 100083, China

**Keywords:** in situ growth, graphene film, sensing performance, transfer process

## Abstract

Graphene films made by chemical vapor deposition (CVD) are a popular method to modify sensors by virtue of large-scale and reproducibility, but suffer from various surface contamination and structural defects induced during transfer procedures. In-situ growth of graphene films is proposed in this review article to improve sensing performance. Root causes of the surface contamination and structural defects are revealed with several common transfer methods. In-situ approaches are introduced and compared, growing graphene films with clean surfaces and few defects. This allows graphene film to display superior sensing performance for sensor applications. This work may reasonably be expected to offer a good avenue for synthesis of graphene films applicable for sensing applications.

## 1. Introduction

Graphene films in chemical vapor deposition (CVD) are known to modify sensors by virtue of large-scale and reproducibility, but suffer from various surface contamination and structural defects induced during transfer procedures. Graphene is a stable two-dimensional atomic film with *sp^2^* hybridized carbon atoms, and possesses unique performance characteristics, such as high carrier mobility, high thermal conductivity and high mechanical strength [1,2,3]. This allows graphene to offer promising applications in the field of sensors [4,5,6,7,8]. Graphene film has less contamination and low defect density. The structures and performance of graphene depend on the respective preparation processes. Surface contaminants and structural defects stem from both growth and transfer procedures of graphene preparation. Some researchers focus on the defects and side reactions during growth. Typical findings include: a high-temperature annealing procedure may repair some of the defective sites [9,10]; the occurrence of side reactions may be reduced by adjusting growth parameters, such as temperature, pressure and carbon source [11,12]. Such methods may only cope with the surface contamination and structural defects in a certain degree. Apart from the growth procedure, the graphene transfer is an indispensable procedure during application, and affects the resulting graphene film [13]. Consequently, in-situ growth of graphene films is proposed to reduce the structural defects and surface contaminations for a better sensing performance. 

In-situ growth refers to direct growth of graphene on the target substrate, and can avoid complex transfer steps and post-processing processes [14]. This may not only significantly optimize procedures of graphene preparation, but also ensure the integrity of the graphene and the consistency of the device [15,16,17]. In this review article, the root causes of surface contamination and defects are illuminated. A comparative analysis is conducted for in-situ growth methods and principles. The application of in-situ graphene in the sensing field is also explored.

## 2. Surface Contamination and Defects Come from Transfer Procedures

The transfer procedure involves transfer of a large-area graphene layer deposited by CVD onto a target substrate. The ideal transfer process is to obtain graphene free of contamination and defects, but actual transfer inevitably leads to the surface contamination and structural defects [18,19]. The residues and contaminants may originate from the sacrificial substrate (e.g., copper foil, silicon wafer); the etchant used to dissolve the substrate (e.g., iron nitrate (Fe (NO_3_)_3_); and the support layer (an organic polymer such as polymethyl methacrylate (PMMA)) [20,21,22,23]. The surface contaminations and defects may be susceptible to cracking and even breakage under mechanical operation during cleaning and transfer procedures [24] and become hidden troubles of future sensing applications [25,26]. 

This section illuminates how the surface contamination and structural defects occur in two commonly used transfer methods and shows their negative effects on the graphene film and device performance.

### 2.1. Wet Transfer

**Figure 1:**
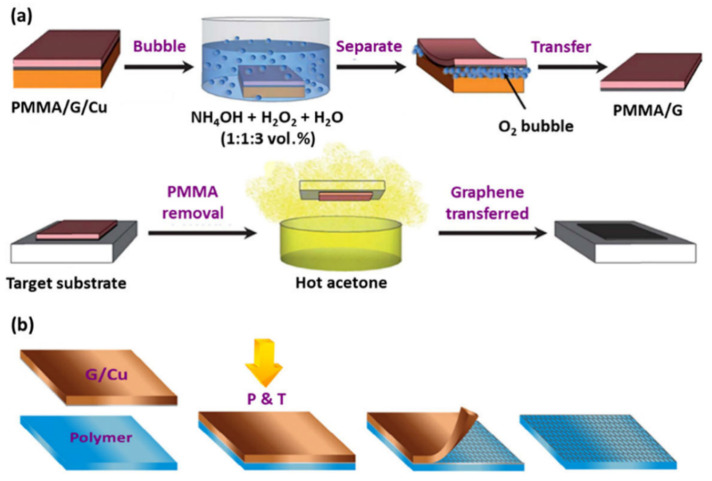
Two commonly-used transfer methods. (**a**) Wet transfer; (**b**) Dry transfer [27].

Photoresist-assisted transfer is the most common-used transfer method. A typical procedure is shown in Figure 1a. A photoresist layer, such as PMMA, is applied on the graphene sample; the substrate is etched with an etching solution; the “PMMA/graphene” stack is transferred to the target substrate; and the PMMA is finally dissolved. The graphene transferred thus can be used for subsequent device fabrication [27]. However, the transfer process is complex and time-consuming, involving multiple steps such as PMMA spin curing, the substrate etching, layer removal, and cleaning. The various steps are also highly susceptible to operational techniques, which makes it difficult to ensure that large-area graphene can be transferred without damage. Even when PMMA is used, structural defects such as wrinkles and cracks are often observed. This problem often occurs in mechanical operations, especially the layer removal, by means of fold gaps or water (including steam) trapped between the graphene and the target substrate. [28,29]. Another problem is residues from removal of the polymer support layer. The residues may harm the electrical performance of graphene electronics (e.g., graphene field effect transistors (FETs)) because they act as barriers between the scattering center of carriers and the metal. Even if the residues of metal particles can be removed in a post-treatment process, this increases the complexity of the preparation process and the production costs and is not advisable for scaled up processes [30].

### 2.2. Dry Transfer

Dry transfer is another commonly-used method. By mean of mechanical exfoliation, dry transfer is popular in sensor applications due to the absence of surface polymer and interfacial water contamination, which lowers the cost. As shown in Figure 1b, the quality of transferred graphene is dependent on the viscosity of the polymer, and a lower viscosity may lead to structural defects. Mechanical transfer inherently requires stronger interfacial interaction between graphene and the target substrate (or support layer) than that of graphene/metal. In fact, it is difficult to fulfill the interfacial interaction uniformly in actual transfer operations, and this in turn leads to graphene being prone to structural defects such as cracks and an uneven surface. The presence of these defects may reduce the tensile strength of the film, and the mechanical performance may become worse with increased numbers of defects [31]. The defects may thus have a serious impact on the lifetime of the device and the associated properties. Notably, the broken areas may introduce air bubbles, and make the adsorption performance between the graphene film and the substrate deteriorate. This may even cause the graphene sensing layer of the device to fall off during sensing applications.

## 3. In-Situ Growth Makes Graphene Film of Fewer Defects Available 

The transfer procedures make graphene film prone to breakage, surface contamination and lattice defects, and directly affect the performance and lifetime of the device [32]. The post-processing goal is always to get graphene films with few defects and clean surfaces. However, the transfer procedures will undoubtedly increase the complexity and cost of the preparation process, and the treated layers will still have difficulty in achieving the pre-transfer properties. In-situ growth of graphene films may be the preferred solution to avoid these drawbacks. In-situ growth may get rid of complex transfer and subsequent procedures and avoid the associated structural and performance defects. In-situ growth methods can be divided into three categories according to the growth mechanism and the catalyst introduced, i.e., metal-free catalytic growth, metal-assisted growth and plasma-enhanced growth.

### 3.1. Metal-Free Catalytic Growth

The key to metal-free catalysis is to achieve the cracking of the carbon source. Without involvement of the catalytic metal, the decomposition of the carbon source becomes rather slow and often takes a long period (several hours or even dozens of hours). Some special experimental parameters have to be employed for preparing graphene, such as high-flux carbon, and high working temperatures (above 1400 °C). In this way, in situ growth of graphene can be achieved by adjusting the preparation steps in the absence of a catalyst.

**Figure 2:**
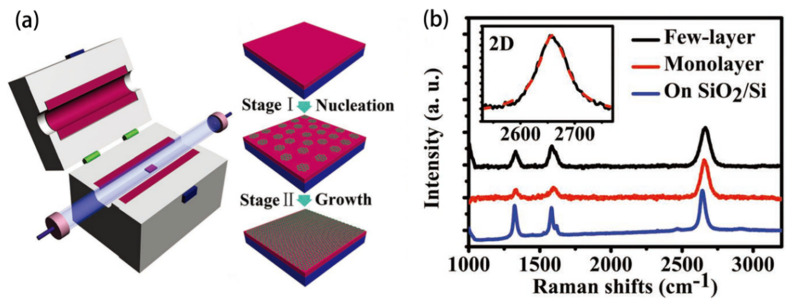
Two-step growth. (**a**) Apparatus and growth mechanism of graphene grown in a two-step method; (**b**) Raman characterization of in situ graphene [33].

A two-step growth technique is reported by Chen [33]. As shown in Figure 2a, the in situ growth can be divided into two steps: graphene nucleation and growth. The nucleation site density and grain size are controlled by adjusting the carbon source concentration, and oligomeric graphene without catalyst. The Raman result in Figure 2b shows that the I_2D_/I_G_ at 2660 cm^−1^ is relatively high (>4), confirming the existence of monolayer graphene, and its electron mobility in air reaches 1510 cm^2^V^−1^s^−1^ in performance tests.

**Figure 3:**
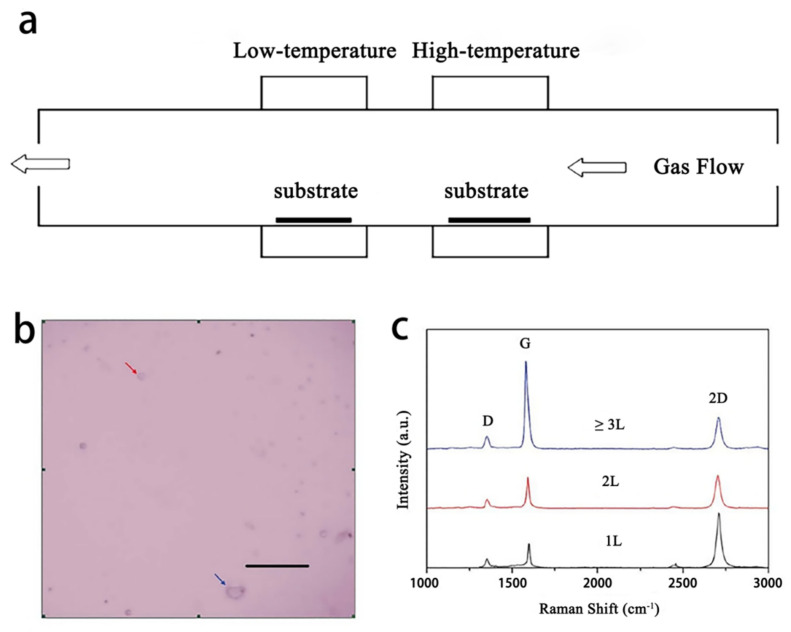
Graphene growth in a tube furnace. (**a**) Schematic diagram of graphene on SiO_2_ substrate, assembled with two temperature zones; (**b**) Light microscope image of the sample after growth in the low temperature zone; (**c**) Raman spectra of three regions corresponding to the blue and red arrow labeled areas as well as the background, showing the presence of single, double, and multilayer graphene. Scale bar: 10 μm [34].

Xu et al. [34] use a tube furnace to grow graphene in a partitioned manner. As shown in Figure 3a, the tube furnace is divided into a high temperature zone and a low-temperature zone; the substrate is placed in the low-temperature zone and the carbon source is introduced from the right end. The carbon source is cleaved as it passes through the high-temperature zone, and then goes through the low-temperature zone to grow graphene on the substrate. Figure 3b,c show the optical microscope images and Raman spectra of the graphene samples, respectively. A combination of Figure 3b,c indicates that single-layer or double-layer graphene may appear, and the high intensity ratio of 2D peak to G peak in Figure 3c proves the existence of single-layer graphene. Moreover, the intensity ratio of D-G is about 0.3 in Figure 3c, and indicates that the prepared graphene has a very low defect density.

### 3.2. Metal-Assisted Growth

Metal-assisted growth is similar to the conventional CVD method in that the carbon source is easily decomposed, and its growth rate is faster under the same conditions due to introduction of a metal catalyst. Under suitable conditions such as a high working temperature (around 1000 °C), the metal-assisted method may produce graphene films with few defects and a uniform layer, favorable for large-scale applications [35]. This method often produces an interface between the metal layer and the substrate, also known as interfacial growth [36].

**Figure 4:**
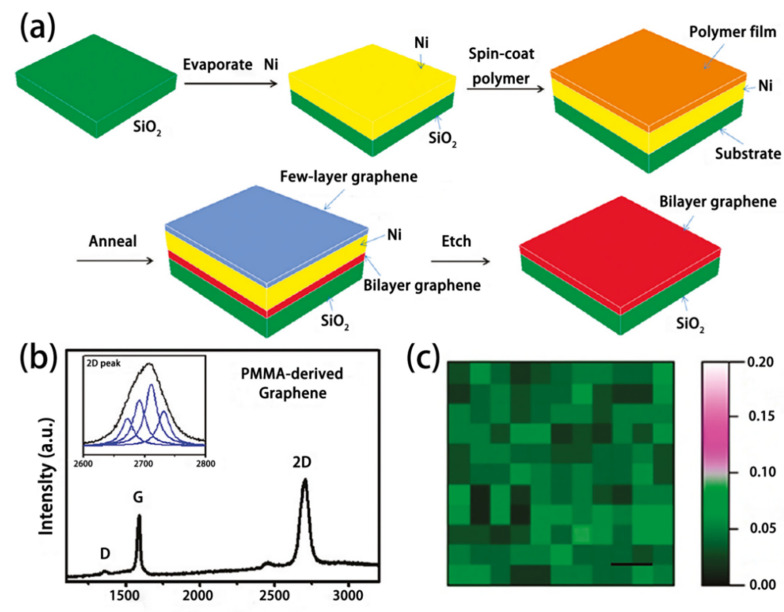
Graphene growth from solid carbon sources. (**a**) growth process; (**b**) Typical Raman spectra of PMMA-derived bilayer graphene; (**c**) D/G peak ratio mapping in area of 100 × 100 μm^2^ for PMMA-derived bilayer graphene [37].

Metal sacrificial layer is a typical example, and the popular catalytic metal is copper or nickel. Such metal can increase the growth rate of graphene and reduce the residual catalytic metal by taking advantage of its catalytic effect and easy removal. Researchers at Rice University report a nickel-catalyzed method [37]. They find that some of the carbon atoms precipitate out of the interface between the nickel layer (400 nm) and the substrate, and graphene grows thereon. Nickel has a high degree of carbon in solid solution, and carbon atoms precipitate out of the nickel layer during the cooling process and generate graphene [37]. As shown in Figure 4a, PMMA is used as a solid carbon source and is spin-coated on the nickel surface after of annealing; a graphene film then occurs at the interface between the nickel layer and the substrate. Subsequently, the nickel layer is etched off, leaving only graphene grown at the interface.

As shown in Figure 4b, a D peak at 1350 cm^−1^ in the Raman spectrum suggests that PMMA-derived graphene films are free from defects or symmetry breaking locations. Furthermore, Figure 4c shows that the peak ratio of D to G is less than 0.1 in most places, and confirms a good quality of the graphene. Laurent Baraton et al. [38] prepared graphene films in a similar growth process, the difference being that the carbon atoms they used were injected into the nickel layer by an ion implantation technique. This allows precise control of the number of carbon atoms and then the layer number of graphene for preparing a homogeneous and controllable graphene film.

**Figure 5:**
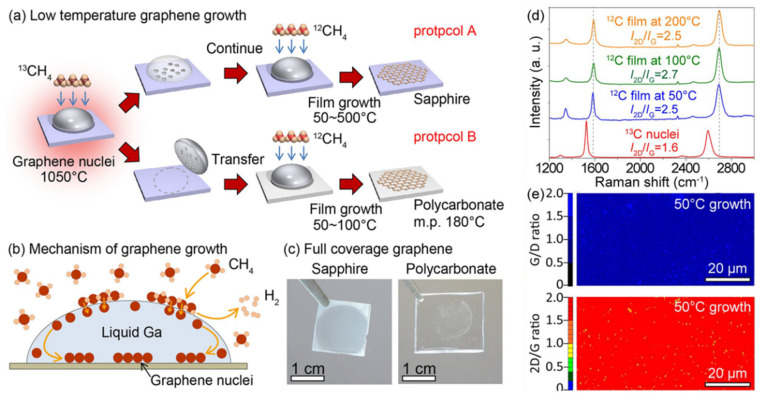
Liquid gallium-catalyzed graphene. (**a**) Schematic diagram of two experiments (A,B) in low temperature; (**b**) Mechanism of graphene nucleation and growth; (**c**) Optical images of graphene films covered on sapphire and polycarbonate substrates; (**d**) Raman spectra of graphene films prepared at 50 °C, 100 °C and 200 °C; (**e**) Intensity ratios of I_G_/I_D_ and I_2D_/I_G_ of samples grown at 50 °C [39].

Researchers at Tsukuba University [39] present a catalyzed method using liquid gallium under ambient conditions, approaching a lower preparation temperature. As shown in Figure 5a,b, methane decomposes carbon atoms on surface of liquid gallium, and the carbon atoms move below the liquid gallium to form graphene at the interface between the liquid gallium and the substrate. The liquid gallium is then blown away with a nitrogen gun and the graphene film remains on the substrate. This method has the advantage of low temperature growth and allows for a wider choice of substrates to be grown. The disadvantage is that the island nuclei have to be formed on the substrate at first, and the graphene nucleation sites can be available in case that the low temperature growth has a good coverage. As can be seen from the optical images in Figure 5c, this method makes uniform and transmissive graphene films available on a polycarbonate substrate, and offers new possibilities to apply graphene film for sensor devices made of plastic.

Figure 5d shows the intensity ratios from the Raman spectra, and the ratios of I_G_/I_D_ and I_2D_/I_G_ indicate that surface defects of graphene are few and affected by the temperature. As shown in Figure 5e, the graphene films are homogeneous and almost free of contaminants on the surface, even at temperatures as low as 50 °C. This suggests that the liquid metal may have a stronger catalytic effect and effectively lower the preparation temperature.

### 3.3. Plasma-Enhanced Growth

**Figure 6:**
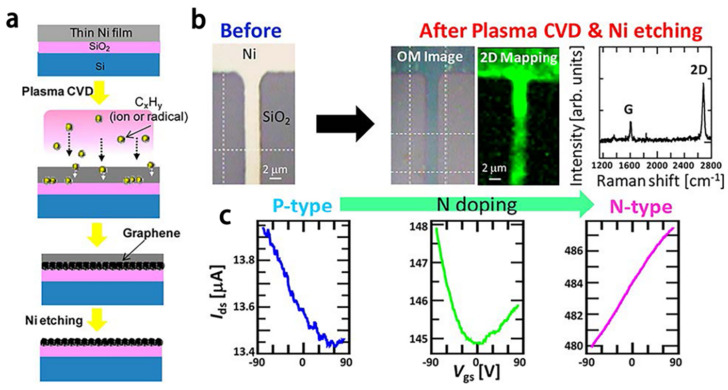
Plasma-enhanced growth. (**a**) Growth mechanism of graphene on the SiO_2_ substrate; (**b**) Optical micrograph of graphene along with Raman spectrum; (**c**) Typical I_ds_-V_gs_ curves for graphene grown at 950 °C, with NH_3_ flow rates of 0 sccm, 5 sccm, and 15 sccm, respectively [40].

Plasma-enhanced growth cracks the carbon source such as methane by means of plasma. Using plasma as a catalyst, the preparation temperature can be lowered (below 800 °C) and a high growth rate can remain. Researchers at Tohoku University in Sendai, Japan [40] jointly use plasma and nickel catalysis to prepare graphene film. As shown in Figure 6a, nickel film (5 nm) is deposited on the substrate. After the gaseous carbon source is ionised by the electric field, the carbon atoms are driven into the nickel film by acceleration of the electric field, and the carbon atoms precipitate out of the nickel film and form graphene at the interface between the nickel film and the substrate after cooling; the graphene film is finally achieved after removing the nickel film. Figure 6b shows that the I_2D_/I_G_ is around 1.8 and the D peak intensity is very low, and indicates that the resulting graphene has few defects. As shown in the typical I_ds_-V_gs_ curve in Figure 6c, the charge neutral point shifts towards the negative gate bias voltage as the NH_3_ flow rate increases. This indicates that the doping concentration can be regulated by adjusting the concentration of the electron donor, which is of importance for construction of electronic devices such as logic circuits for sensors.

Similar to the ‘two-step’ method in the metal-free catalytic approach described above, researchers at the National University of Singapore used a plasma-enhanced chemical vapor deposition (PECVD) system to prepare graphene film on insulating substrates at low temperatures [41]. Graphene film is achieved on insulating substrates at low temperatures in a ‘two-step’ method. This method can be used in the case that the mechanism separates graphene nucleation with growth. With the aim of reducing the nucleation site density of graphene, the single crystal area of graphene can be increased to reduce the surface contamination and structural defects.

**Figure 7:**
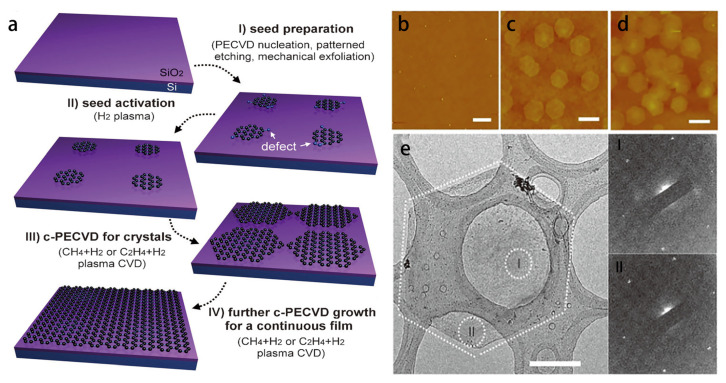
Graphene growth in a remote radio frequency (RF) PECVD system. (**a**) Graphene preparation flow; (**b**) AFM images of graphite agglomerates after nucleation at 650 °C; (**c**,**d**) AFM images of graphene on SiO_2_/Si after c-PECVD (CH_4_ + H_2_) growth at 600 °C for 90 min (**c**) and 120 min (**d**); (**e**) TEM image of graphene highlighted with a dashed line. The two images on the right are electron diffraction images of selected areas in the region shown by the dashed line [41].

As shown in Figure 7a, the growth is divided into two steps, graphene nucleation and graphene growth. The graphene nucleation sites are obtained at a high temperature (650 °C), and a graphene film is grown a lower temperature (600 °C). With involvement of the plasma, the growth temperature is lower as compared to the metal-free catalytic method. They also introduce hydrogen plasma to etch the edges of the graphene nucleation sites for activation. Figure 7b–e show AFM (Atomic Force Microscope) Figure 7b-d and TEM (Transmission Electron Microscope) Figure 7e of graphene film at different growth times, and show that this method allows clean and less defective single crystals for the graphene film. The problem with this method is the difficulty of preparing large-area graphene films.

### 3.4. Comparison of In-Situ Growth Methods

Table 1 lists advantages and disadvantages of the three in-situ growth processes as compared with the conventional CVD method. Metal-free catalytic growth is a typical in-situ growth method, and has a unique advantage applicable to various large-area substrates with morphologies. The substrate morphologies can be rough, curved, or three-dimensional, which is not applicable for CVD, or most metal-assisted growth methods. This method allows graphene modification to construct sensor devices on different substrates in a simple substrate pretreatment. The method is subject to two disadvantages. One is the long growth period (several hours or even dozens of hours) because of the absence of a catalytic metal. Another is the high working temperature (about 1400 °C), which is unacceptable for some metal substrates.

Metal-assisted growth has virtue of less contamination and defects in the graphene and good homogeneity with the help of catalytic metals. Under suitable growth conditions, the growth rate can reach 3 to 4 times that of the metal-free catalytic method. Controlled growth of graphene can also be achieved by using metal film with high catalytic activity, and the graphene can be grown with a set number of defects, film layers and doping levels. This growth also has two disadvantages. One is the complex and high-cost process. Another is that the catalytic metals may contaminate the substrates; especially for the substrates of semiconductors, the metal atoms may be doped into the semiconductors and lessen the device performance.

Plasma-enhanced growth has a high growth rate and a low working temperature with help of plasma. This method has two disadvantages. One is poor controllability. Another is the difficulty of preparing a homogeneous graphene film with large area. 

In brief, there has been much progress in in-situ growth of graphene films, but there is still a long way to go before there can be large-scale promotion and application of in-situ graphene in the field of sensing.

## 4. Use In-Situ Grown Graphene Film to Improve Sensing Performances

Graphene presents a promising application potential in the field of sensing due to its unique planar structure and superior performance as compared with other sensing materials [42,43,44,45]. In-situ growth may enable the graphene film to avoid surface contaminations and defects, and facilitate its applications in sensing fields such as electrochemistry and optics [46,47].

### 4.1. In-Situ Graphene Modified Self-Supported Boron-Doped Diamond Electrode for Pb (II) Electrochemical Detection in Seawater

Detection of heavy metal ions in the offshore environment has been a matter of intensive concern since Minamata disease occurred in Japan [48,49]. Pb^2+^ stealthily affects human health through the action of the food chain, and leads to life-threatening situations. Real-time monitoring of Pb^2+^ content in seawater is a primary approach to prevent risk associated with Pb^2+^ [50,51,52]. We propose using a metal-catalyzed in-situ grown graphene film to develop a graphene modified self-supported boron-doped diamond electrode (G/SBDD) to detect Pb^2+^ in seawater [53]. Compared with the unmodified SBDD (Self-Supported Boron-Doped Diamond Electrode), G/SBDD enables the detector to display both superior sensing performance (high conductivity, high electrochemical active area) and detection performance (high sensitivity, low detection limit, high selectivity).

**Figure 8:**
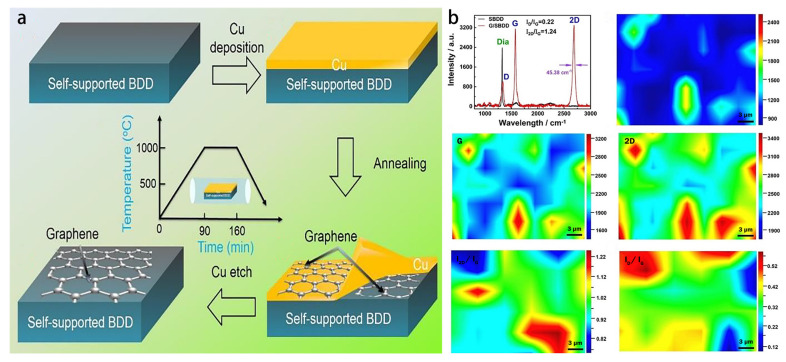
(**a**) Schematic diagram of G/SBDD preparation process; (**b**) Raman spectra of SBDD and G/SBDD.

As can be seen in Figure 8a, the G/SBDD sample preparation process is divided into four steps. Firstly, the SBDD sample was prepared using hot filament chemical vapor deposition. The sample was cleaned by ultrasonication in acetone solution to remove surface contaminants, dried and placed in the magnetron sputtering chamber. Next, a copper film was deposited on the SBDD surface by magnetron sputtering. Afterwards, the Cu/SBDD samples were annealed in a vacuum annealing furnace to create the Cu-Graphene-SBDD structure. Finally, the Cu film on the annealed sample surface was etched to obtain the G/SBDD sample. The introduction of a high-temperature annealing process may lead to a more stringent selection of growth substrates, limiting the in-situ growth and application of graphene in a wide range of substrate materials.

The number and quality of layers of in situ modified graphene on the SBDD surface directly affects the sensing performance of the Pb^2+^ ion sensor, and information on the generation of graphene on the G/SBDD surface was characterized using Raman spectroscopy. Figure 8b illustrates the SBDD and G/SBDD Raman spectra. It can be seen that the characteristic peaks of the graphene phase on the G/SBDD surface are significant and the quality of graphene production is high. For G/SBDD, a D peak at ~1350 cm^−1^, a G peak at ~1580 cm^−1^ and a 2D peak at ~2700 cm^−1^ can be observed. The G-peak is a typical characteristic peak of graphene and the presence of the high intensity G-peak in this experiment demonstrates the successful modification of graphene. The 2D-peak is the second order Raman peak of graphene and the number of layers of graphene is determined by the FWHM value of the 2D-peak and the ratio of D-peak to G-peak. The FWHM (Full Width Half Maximum) value of the 2D peak was 45.38 cm^−1^ and the I_2D_/I_G_ value was 1.24; it was shown that when the FWHM of the 2D peak was in the range 45–60 cm^−1^ and the I_2D_/I_G_ value was in the range 0.7–1.3, the resulting graphene was a bilayer graphene. The I_D_/I_G_ was as low as 0.22, indicating that the defect content of the graphene was low and the quality of production was high; this also shows that the high temperature annealing was able to achieve partial defect healing of the graphene. Furthermore, it can be demonstrated that the SBDD surface was successfully modified in situ with high quality bilayer graphene [54].

**Figure 9:**
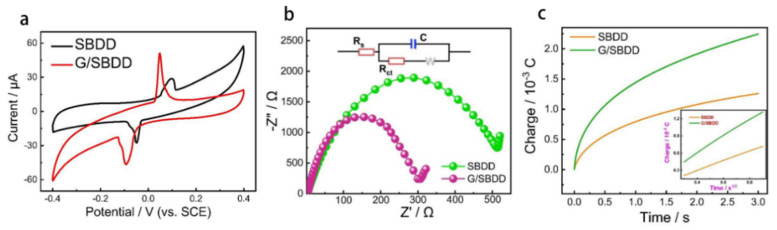
Characterization results of the sensing performance of G/SBDD and SBDD. (**a**) CV curves of SBDD and G/SBDD electrodes in 0.1 M Na_2_SO_4_ solution containing 5 ppm Pb^2+^ with a scan rate of 10 mV/s; (**b**) Nyquist plots of SBDD and G/SBDD electrodes in 0.1 M Na_2_SO_4_ solution containing 5 ppm Pb^2+^ with a frequency range of 10^−2^ to 10^5^ Hz; insets are equivalent circuit diagram; (**c**) Q-t curves of SBDD and G/SBDD electrodes in 0.1 M Na_2_SO_4_ solutions containing K_3_ [Fe (CN)_6_]; inset shows linear partial charge (Q) as a function of t^1/2^.

The reaction kinetic transition characteristics of Pb^2+^ on the surface of the SBDD and G/SBDD are important criteria to measure whether the in-situ graphene modification can enhance the sensing performance, and cyclic voltammetry is an effective way to analyze the reaction kinetics. The test results showed that the peak value of Pb^2+^ on G/SBDD was higher than that on SBDD (Figure 9a). The increase in the peak value of Pb^2+^ indicated that the in-situ modification of graphene improved the electrical conductivity of SBDD, resulting in a good conductive circuit between the graphene sensing layer and the solution system, providing more electronic pathways for the transfer of Pb^2+^.

To further investigate the effect of in situ modification of graphene on the SBDD electrode surface on the charge transfer capability between the sensing platform and the solution system, a relevant circuit was constructed in a 0.1 M Na_2_SO_4_ solution of 5 ppm Pb^2+^ for EIS testing. The Nyquist plot in Figure 9b shows that the charge transfer resistance of the SBDD electrode was 546 Ω, while that of G/SBDD was 312 Ω. The reduced charge transfer resistance further indicates that the in-situ modification of graphene reduces the charge transfer resistance at the electrode surface, accelerates the charge transfer rate and improves the responsiveness of the device to Pb^2+^.

The electrochemically active area of the electrode is the area of the active site where the probe binds and reacts with the probe ion, and is an important metric for evaluating the sensing performance of the device. In general, the larger the electrochemically active area, the better the sensing performance of the device and the better the modification effect exhibited by graphene. The group measured the electrochemically active area of in situ grown graphene-modified G/SBDD electrodes and unmodified SBDD electrodes, respectively. They used K_3_ [Fe (CN)_6_] as the redox probe and Na_2_SO_4_ solution as the electrolyte to determine the electrochemically active area of the devices by the chrono-coulometric method. The experimental results are shown in Figure 9c. A higher number of charges were adsorbed on the surface of the G/SBDD electrode compared to the SBDD electrode in the same time, indicating that the G/SBDD electrode was able to provide more reactive sites. At the same time, the test results showed that the electrochemically active area of the G/SBDD was up to 2.7 cm^2^, which was much higher than the measurements obtained for the SBDD under the same process (1.6 cm^2^), and the in-situ graphene modification increased the active area of the electrode by a factor of almost 1.7. The increase in the active area of the electrode is due to the in situ modified graphene film expanding the contact area of the electrode with the electrolyte, which also provides more binding and reaction sites for the redox probe.

In the process of exploring the detection performance of the sensor, it is necessary to focus on the analysis of important indicators such as the standard curve, sensitivity and detection limit of the sensor. The calibration curve shows the linear relationship between the ion concentration and the peak current value of the dissolved ion, and provides a visual representation of the concentration of the detected ion. The degree of change of the dissolved signal with increasing concentration reflects the sensitivity of the detection, and a high sensitivity helps to improve the detection accuracy of the electrode in the low concentration range. The detection limit is the lower limit of detection of the sensor and is an important indicator for determining the effective working range of the sensor [55].

**Figure 10:**
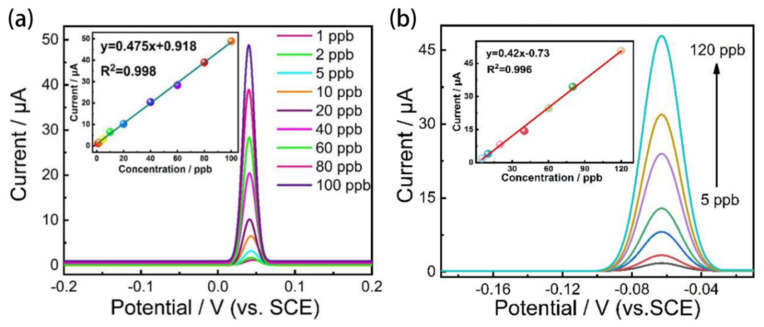
Standard curves and detection limits for G/SBDD and SBDD. (**a**) Dissolution curve of G/SBDD electrodes for Pb^2+^ at concentrations ranging from 1 to 100 ppb; inset shows the fitted curve of ion concentration versus dissolution peak intensity; (**b**) Dissolution curves of SBDD electrodes for Pb^2+^ at concentrations ranging from 5 to 120 ppb; inset shows the fitted curve of ion concentration versus peak dissolution current value.

As can be seen from Figure 10a, the ion concentration maintained an excellent linear relationship with the dissolved peak current value over a linear range of 1 to 100 ppb. As shown in the calibration curve of G/SBDD in the inset of Figure 10a, the sensitivity of the G/SBDD electrode reached 0.475 μAL μg^−1^ cm^−2^, while the sensitivity of the SBDD under the same process conditions was only 0.42 μAL μg^−1^ cm^−2^ (shown in Figure 10b). Further calculating the detection limit of the G/SBDD electrode using 3N/S (N is the electrode noise value; S is the electrode sensitivity), it can be obtained that the detection limit of the G/SBDD sensor for Pb^2+^ is approximately 0.21 ppb, which is nearly five times lower than the minimum permissible concentration of Pb^2+^ in seawater specified in GB 3097-1997. This is also nearly five times lower than the detection limit measured by the SBDD (1.02 ppb), demonstrating that the in-situ modification of graphene has brought about a significant improvement in detection performance in terms of detection accuracy and detection limits. The enhancement effect of graphene modification on detection performance may be attributed to the superior adsorption capacity and outstanding electronic transmission capacity of graphene. On the one hand, the superior adsorption capacity endows G/SBDD electrode with a stronger enrichment effect on Pb^2+^, and brings a lower detection concentration than that of SBDD. On the other hand, the outstanding electronic transmission capacity of graphene could significantly facilitate the mass transfer process of the G/SBDD electrode, thus inhibiting the intermediates from participating in the reaction process. The above two facts jointly allow the G/SBDD electrode to acquire a smaller noise and provide a significant improvement on detection limit.

Reproducibility and selectivity are important indicators to determine whether the sensor can maintain a stable performance in practical applications. Reproducibility reflects the degree of decay of the detection signal with an increasing number of detections and is an evaluation indicator of the accuracy of the sensor; while selectivity reflects the ability of the sensor to resist interference ions. To investigate the reproducibility of the G/SBDD electrodes, the group performed nine reproducibility experiments. The electrolyte used was a 0.1 M Na_2_SO_4_ solution containing 20 ppb Pb^2+^, and six interfering ions, Ca^2+^, Mg^2+^, Zn^2+^, Fe^2+^, Cu^2+^ and Cd^2+^, were added to the electrolyte to simulate the complex detection environment. The content of the interfering ions was the same as that of Pb^2+^, all at 20 ppb, and a constant potential of +0.3 V was applied to the electrode at the end of each measurement and held for 100 s to avoid interference with the experiment by residual metal on the electrode surface.

**Figure 11:**
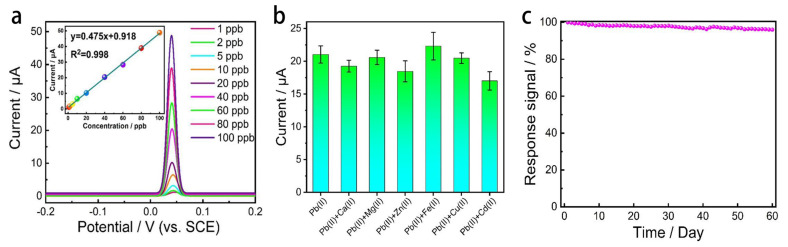
G/SBDD reproducibility, selectivity and thermoregulation. (**a**) Dissolution curves of Pb^2+^ from nine replicate experiments on G/SBDD electrodes; (**b**) Effect of the six interfering ions on the Pb^2+^ dissolution signal from the surface of the G/SBDD electrode; (**c**) Signal response of G/SBDD electrodes as a function of time.

The dissolution curves obtained from the nine replicate experiments are shown in Figure 11a. The peak dissolution potential of Pb^2+^ remained constant with increasing number of experiments; the peak current values decreased slightly, but the overall decay was low. The relative standard deviation (RSD) of the Pb^2+^ dissolved peak current values obtained from the nine replicate experiments was calculated and the RSD values were used to represent the accuracy of the electrode. The calculated RSD value of 2.7% for the nine replicate experiments for Pb^2+^ indicates that the G/SBDD electrode has good reproducibility and reflects the high accuracy of the graphene in situ modified device. From the interference test results shown in Figure 11b, it can be seen that the addition of the interfering ions did not cause any drastic interference to the Pb^2+^ dissolution signal, and the Pb^2+^ dissolution signal did not change significantly with the addition of Zn^2+^, Fe^2+^, Cu^2+^ and Cd^2+^. This indicates that the G/SBDD electrodes are more resistant to the above-mentioned heavy metal ions and the inherent metal ions in the water column, demonstrating the good selectivity of the in situ modified Pb^2+^ sensor.

In addition, the G/SBDD electrodes were immersed in a solution of 30 ppb Pb^2+^ in 0.1 M Na_2_SO_4_ for 60 days for stability testing. The solution was stirred to simulate the flow of water and the Pb^2+^ content was measured daily using the electrode. Figure 11c shows the signal response of the G/SBDD electrode as a function of time. The response of the electrode after 60 days is 96% of the initial value, demonstrating the stability of the device and the difficulty of stripping or contaminating the graphene sensing layer, which effectively guarantees the lifetime of the sensor.

Systematic analysis and performance comparisons show that the G/SBDD with in situ graphene modification exhibits superior sensing and detection performance compared to the unmodified SBDD electrode. In addition, it has great advantages in terms of device structure, as in-situ graphene modification can significantly improve the device preparation efficiency and device uniformity. It also effectively solves the problem of easy detachment of the sensing layer, which often occurs in transfer graphene modified sensors, and improves the anti-interference capability and service life of the device, which provides a strong guarantee for the normal service of the device in complex detection environments.

### 4.2. Infrared Sensor Based on Graphene-Silicone Schottky Contact for Earth Observation Mission

Earth observation is a description of the current environmental conditions and conditions of human existence, which can provide valuable information to help human beings know the earth’s conditions more clearly. Among them, infrared sensors are commonly used in non-contact temperature measurement, gas composition analysis and nondestructive testing, which are widely used in the field of environmental engineering in earth exploration [56].

Recently, the research team of the Institute of Materials Science of Kaunas University of Technology (KTU) developed a new infrared sensor based on graphene-silicone Schottky contact. This new infrared sensor can help us to observe the Earth and carry out interplanetary missions from space to explore the atmospheres of other planets or look for life on Mars. Dr. Šarūnas Meškinis, the lead researcher of the research team, pointed out that the manufacturing technology for the Schottky contact sensor is much simpler than for other infrared sensors. Multiple arrays of this sensor can be developed on semiconductor boards (for example, planar silicon boards). Stable structure and simple process are the main advantages of the Schottky contact sensor [57]. However, this Schottky sensor has a serious disadvantage, that is, the responsiveness and sensitivity of the device are relatively low [58]. The main reason is that the conventional transfer graphene film is limited by its light absorption rate of only 2.3%, so it can only convert a small number of optical particles into photoelectrons, making it difficult to obtain a large sensor responsiveness.

**Figure 12:**
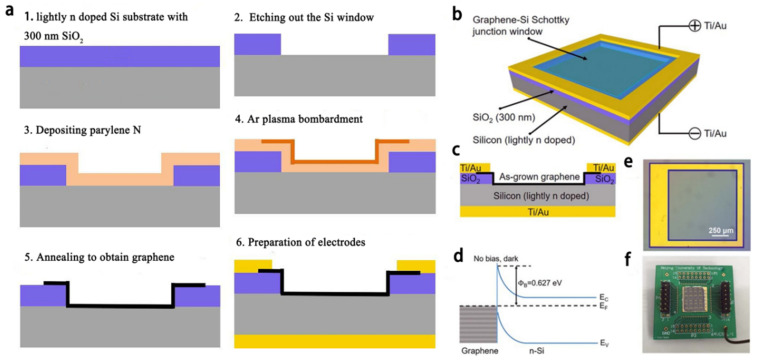
(**a**) Construction process of in-situ grown graphene-silicon Schottky junction photoelectric sensor. (**b**) Schematic diagram of the structure of the device. (**c**) Schematic diagram of cross-section structure of the device. (**d**) Schematic diagram of energy band structure of Schottky junction. (**e**) Optical microscope image of the device. (**f**) Physical drawing of the device.

Based on this, scientists have proposed a Schottky junction photoelectric sensor modified by graphene grown in situ without metal catalysis [59,60]. Different from the construction process of conventional graphene devices, it adopts the process of first preparing electrodes and then growing the graphene. Through the analysis of relevant experimental data, it is found that repeated lithography will affect the morphology of graphene. Therefore, in order to reduce the photolithography process steps after growing graphene, the process of preparing the electrode first is adopted, which can effectively avoid the melting of the electrode and reduce the diffusion of carbon atoms in the substrate. Figure 12a shows the fabrication process of the device. Based on the metal-free catalytic in-situ growth technology mentioned above, SiO_2_ lightly doped silicon substrate is selected as the growth substrate, and then the graphene growth window is etched on the surface of the sensor substrate by photolithography. In addition, in order to reduce the diffusion of carbon atoms in the substrate, it is necessary to set the growth temperature to a lower temperature. Finally, the sample is placed on the circuit board of the sensor, the graphene fixed to the electrode by silver glue, the edges of the silver glue and graphene are protected by silica gel to prevent short-circuit leakage of the device, and the device is finished.

**Figure 13:**
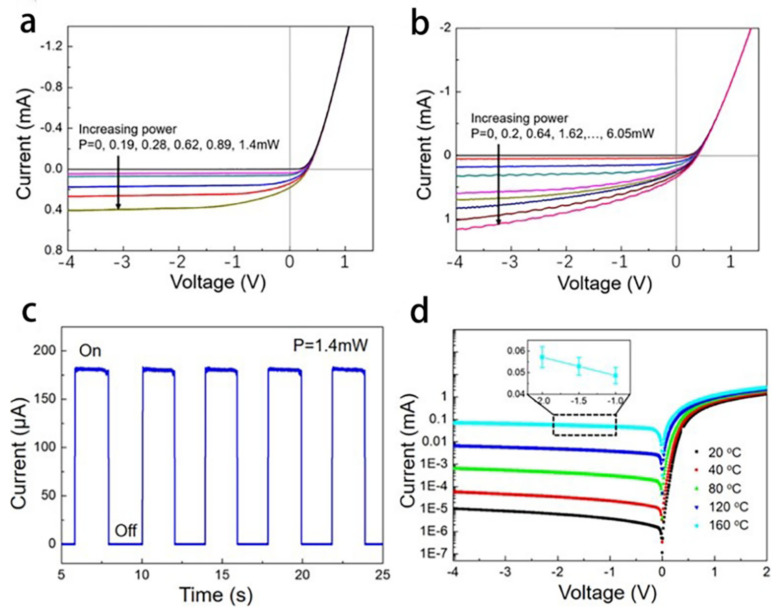
Photoelectric performance test results of in-situ graphene-silicon Schottky sensor. (**a**,**b**) I-V characteristic curves at different incident light powers. (**a**) White LED light source. (**b**) 792 nm laser light source. (**c**) The light response of the device within five switching periods under the irradiation of a white light source. (**d**) I-V characteristic curves of dark field characteristics of the device at different working temperatures. The illustrations amplify the current of −2V~ −1V when the device works at 160 ℃.

The device structure is shown in Figure 12b,c. Figure 12d shows the schematic diagram of the energy band structure of the device. Figure 12e shows the optical microscope image of the device, and Figure 12f shows the physical picture of the graphene device. It can be seen from the above figure that the in-situ graphene-silicon Schottky sensor has been successfully constructed. Then, the performance test was carried out. In the performance test, white light emitting diode (LED) light source and near infrared (792 nm) laser light source were used, and the I-V characteristic curves of the device were obtained under different powers of incident light (as shown in Figure 13a,b). From the figure, it can be found that when the incident light power of white LED is 0.19mW, the response of the device under zero bias voltage is 206 mA/W. When the incident optical power of 792 nm laser is 0.2 mW, the responsivity of the device is 205.7 mA/ W under zero bias, 267.3 mA/W under 2V reverse bias and 275.9 mA/W under 4 V reverse bias. Figure 13c shows the transient response of the device with 792 nm laser light source under 0V bias voltage. It shows that the response speed in the experiment is less than the test limit of the device (1 ms), which further proves that the device has a very fast response speed. Figure 13d is the I-V characteristic curve of the device in a dark field environment at different operating temperatures. It can be seen that the reverse current of the device increases obviously with the increasing of operating temperature. Therefore, it can be found that the current transport mechanism of the Schottky junction is mainly thermionic emission mechanism.

The performance and structure of the Schottky photoelectric sensor constructed by in-situ growth of graphene film are compared with those of graphene-silicon photoelectric sensors reported in the previous literature (as shown in Table 2). From the performance point of view, the in-situ graphene sensor is more responsive than the silicon Schottky junction sensor constructed by ordinary single-layer transfer graphene film. In addition, it also has a greater performance advantage. If the in-situ grown graphene film is used to construct the device, this greatly improves the device preparation efficiency and device consistency, and the device technology is compatible with the traditional semiconductor technology, which provides favorable conditions for its wide application in the sensing field.

### 4.3. The Graphene Modified Blue LED Sensor

The invention of white LED lamps has greatly improved lighting efficiency, and is known as “the second lighting revolution after Edison”. The mature development of white LED lights began in 2014. Isamu Akasaki, a professor at Nagoya University and Meijo university, Amano Hiroshi, a professor at Nagoya University, and Shuji Nakamura, a professor at the University of California, USA, invented high-brightness blue light-emitting diodes [61]. This enables people to gather LEDs and to emit three primary colors of light, and makes even brighter white light available.

At present, the transparent electrode material commonly used in blue LED sensors on the market is mainly ITO (Indium Tin Oxide), which is used to improve the current spreading on the surface of the LED. As a rare metal, indium is a non-renewable resource, which leads to the high price of ITO material, and hence, the application range of blue LED sensor is greatly limited. While carbon is abundant in the earth, graphene also has good light transmittance and conductivity, so it is an excellent material to replace ITO. The in-situ growth temperature of graphene on gallium nitride (GaN) surface should not be too high, because this would destroy the epitaxial structure of LED [62]. Therefore, the low-temperature growth method, a new in-situ graphene growth technology described above, was adopted, and cobalt was selected as the material for the metal sacrificial layer. Cobalt has strong catalysis similar to nickel, and the quality of graphene grown at low temperature is effectively guaranteed. In addition, the diffusion rate of carbon atoms on cobalt surface is higher than that of nickel, which also ensures the relative uniformity of graphene layers. Although Cu-Ni alloy has good in-situ growth effect, it needs to undergo an annealing process at 600 °C for 40 min before growth. Because the long annealing process would destroy the chip structure of LED, Cu-Ni alloy was not used as catalyst.

The growth of graphene needs to be catalyzed by cobalt metal. At the same time, graphene also acts as the current spreading layer of p-GaN (P-type Gallium Nitride). Therefore, cobalt can be used as both the etching mask of p-GaN mesa and the catalyst of graphene, which can greatly reduce the process steps. Tang et al. [63] put forward the whole preparation process of graphene modified LED photoelectric sensor by cobalt catalysis: (1) Preparing GaN LED substrate; (2) preparing a cobalt thin film on the surface of the substrate by the process of photoetching, sputtering and stripping; (3) etching GaN by using cobalt as a mask to expose n-gan; (4) growing graphene—because the substrate has no catalytic effect, graphene only grows on the surface of the cobalt and keeps the same shape as the p-GaN mesa; (5) spin coating PMMA and drying; (6) etching the sacrificial layer of the cobalt film to make graphene fall on the GaN substrate, and using marble etching solution for cobalt etching; (7) removing PMMA; (8) N electrode and P electrode preparation.

**Figure 14:**
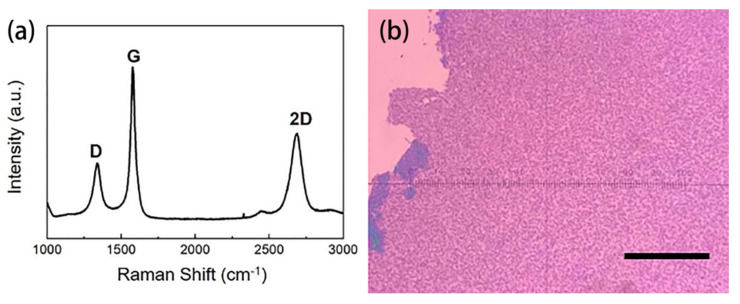
(**a**) Raman spectra of graphene grown in situ catalyzed by cobalt. (**b**) The optical image of graphene grown in situ catalyzed by cobalt, with a scale of 30 μm.

In the traditional GaN device process, metal such as SiO_2_ or Ni is usually used as the mask for etching GaN [64,65]. GaN etching can also be realized by using cobalt film as mask. However, some experimental data show that the Raman signal of graphene on GaN substrate is noisy, which makes the characteristic peak difficult to observe. Therefore, SiO_2_/Si substrate was prepared, and graphene was grown in situ under the same conditions to characterize the quality of graphene. Graphene is grown on the surface of cobalt, so from the growth mechanism, the SiO_2_/Si substrate or GaN substrate should have no influence on the quality of the graphene. Figure 14a shows the measured Raman signal of the graphene. It can be seen that graphene grown at 600 °C catalyzed by cobalt had a small D peak. The D/G peak ratio was about 0.38, and G/2D peak ratio was about 1.8. Figure 14b is an optical picture of the graphene, which can be seen to be significantly thicker than conventional Cu-Ni alloy.

**Figure 15:**
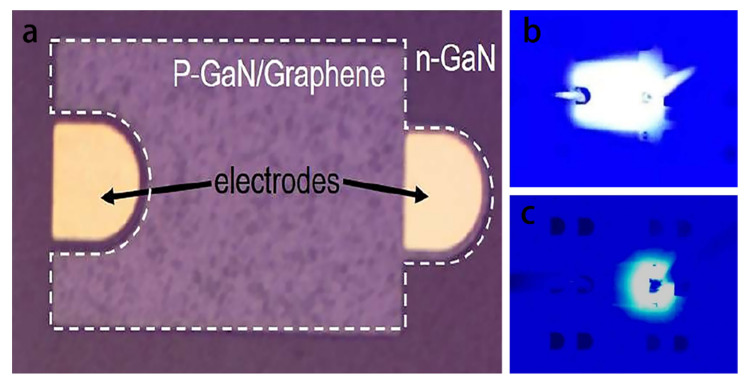
(**a**) Physical diagram of graphene transparent conductive layer GaN LED device. (**b**,**c**) Comparison of luminescence of GaN LEDs with and without graphene: (**b**) Luminescence with graphene; (**c**) No graphene.

Figure 15a is a physical diagram of the LED device, in which the white square area is the p-GaN mesa of the device with graphene film on it. The golden area in the figure shows the electrodes at both ends of the pn junction of the LED, the P electrode on the right and the N electrode on the left. Figure 15b,c show the light emission of the LED with or without graphene. There is a graphene transparent conductive layer in Figure 15b with uniform light emission, and the entire LED device p-GaN mesa is lit. On the other hand, the device of Figure 15c has no graphene, only the area near the P electrode is lit, and the current spread is uneven. In addition, the spectrum of LED grown with graphene is obviously broadened. The luminous flux of the device was tested. Under the working current of 20 mA, the luminous flux of the LED sensor with graphene was 0.135 lm, while that of the LED device without graphene was 0.096 lm. Graphene film improved the expansion of current and increased the luminous flux of the LED sensor by 40.6%.

The above results demonstrate that in-situ grown graphene has an obvious modification effect, and the performance of LED devices is thus improved. At present, the quality of graphene still needs to be further improved, and its conductivity is still far behind that of traditional ITO. In this way, doping may be considered to improve the conductivity. In addition, how to reduce the contact resistance between graphene and GaN is still an urgent problem. Graphene and GaN have large contact resistance, and limit the improvement of device performance. Intercalation between graphene and GaN may be tried to improve the contact resistance.

## 5. Conclusions and Outlook

This paper reviews preparation methods of in-situ grown graphene films and their modification techniques to overcome drawbacks of surface contamination and structural defects that are prevalent in the transfer process. Three in-situ methods are metal-free catalytic growth, metal-assisted growth and plasma-enhanced growth. In-situ growth enables the graphene films to share clean surfaces and few defects. Moreover, this approach may get rid of complex transfer and post-cleaning steps, and facilitates in-situ functional modification of the devices. The application of in-situ graphene films is also explored in the sensing field.

There has been much progress in developing in-situ grown graphene films, and some efficient and feasible methods have been made available. Research on the sensing applications is still in the exploratory stage, and the in-situ growth techniques developed so far have a long way to go before the large-scale promotion and application in the sensing field. The output of this work may serve as a good inspiration and reference for the large-scale development of in-situ graphene films for sensing applications.

## Figures and Tables

**Table 1:** Comparison of advantages and disadvantages of graphene growth methods.

Growth Method	Advantages	Disadvantages	Reference
CVD method	Mature technology Large-area film Good controllability	Complex transfer process	[18,19,20,21,22,23,24,25,26,27,28,29,30,31,32]
Metal-free growth	No substrate selectivity	Long period High working temperature Poor quality	[33,34]
Metal-assisted growth	Fast growth Good quality Graphical growth	Complex and high-cost Metal pollution	[35,36,37,38,39]
Plasma-enhanced growth	Low working temperature	Poor controllability	[40,41]

**Table 2:** Comparison of Silicon-Graphene Schottky junction Photoelectric Sensors.

Device structure	Material	Responsivity	Specific Detection Rate	Light Source
Graphene/planar silicon	Grown in situ	267.3 mA/W (−2Vbias voltage)	4.93 × 10^9^	792 nm laser
Graphene/planar silicon	Transferred graphene	225 mA/W (−2Vbias voltage)	7.69 × 10^9^	488 nm laser
Graphene/planar silicon	Transferred graphene	3.259 μA/KGy cm^2^ (1Vbias voltage)	_	γray
Graphene/planar silicon	Transferred graphene	83 A/W (10Vbias voltage)	10^8^	1.55 μm laser
Graphene oxide/silicon nanowires	Transferred graphene	9.0 mA/W (1Vbias voltage)	_	10.6 μm laser

## Data Availability

Data is contained within the article.
